# Resignation of Prof. Hare

**Published:** 1847-07

**Authors:** 


### RESIGNATION OF PROFESSOR HARE.

We announced, a short time since, the resignation of Prof. Warren, and have now to announce that Prof. Hare has followed his example. Dr. Warren was long one of the ornaments of the Medical School of Boston, and Dr. Hare has for many years contributed his full share to the reputation of the University of Pennsylvania. They have retired from their stations with faculties unimpaired by age, and carry with them into their retirement the respect and good wishes of their colleagues and numerous pupils. It is about forty years since Dr. Hare made himself known to the world by the invention of the *oxy-hydrogen blow-pipe* and important modifications in the galvanic battery, and he has ever since ranked as the first of American chemists. His love for the science has amounted to a passion, and for nearly half a century, in summer and winter, he has devoted himself to it with unvarying zeal. The Board of Trustees of the University of Pennsylvania passed the following resolutions in accepting his resignation:

"Resolved, that in accepting the resignation of Dr. Hare, after an uninterrupted connection of twenty-nine years, the board cannot refrain from expressing to him their high regard for his character, their deep sense of the eminent services which he has rendered to science and to the university, and their earnest wishes for his future happiness.—Resolved, that the appointment of Emeritus Professor of Chemistry be conferred upon Dr. Hare."

